# Clinical Decision-Making in Practice with New Critical Care Ultrasound Methods for Assessing Respiratory Function and Haemodynamics in Critically Ill Patients

**DOI:** 10.3390/clinpract12060102

**Published:** 2022-11-25

**Authors:** Stefan Schmidt, Jana-Katharina Dieks, Michael Quintel, Onnen Moerer

**Affiliations:** 1 Department of Anesthesiology, Emergency and Intensive Care Medicine, University Medical Center, Georg-August-University Göttingen, Robert-Koch-Str. 40, 37075 Göttingen, Germany; 2 Department of Pediatric Cardiology and Intensive Care Medicine, University Medical Center, Georg-August-University Göttingen, Robert-Koch-Str. 40, 37075 Göttingen, Germany

**Keywords:** critical care, critical care monitoring, glycogen storage disease type II, heart transplantation, intracranial pressure, ultrasound, echocardiography

## Abstract

Situations often arise in intensive care units (ICUs) for which only sparse primary evidence or guidelines are applicable or to which existing evidence cannot be applied owing to interactions of multiple disease states. To improve and guide intensive care management in complex scenarios, ultrasonography and echocardiography are invaluable. In five clinical scenarios involving acute deterioration, serial ultrasound examinations of the respiratory system, general critical care ultrasound (GCCUS), and non-invasive haemodynamic critical care echocardiography (CCE) were used routinely. Ultrasonographic results were used to guide further management and initiate experimental therapy or transition from curative to supportive care. The process of initiation of ultrasound examinations to clinical decision-making in these complex scenarios is outlined. These case vignettes highlight the utility of ultrasound and echocardiography. When clinical management is not clear, or evidence is not available, the use of ultrasound for the evaluation of the respiratory system, GCCUS, and non-invasive haemodynamic CCE can help to guide management, reveal newly developed pathologies, lead to clinical management changes, and support the decision for employing experimental therapy approaches in a dynamic way of which few other imaging modalities or monitoring tools are currently capable.

## 1. Introduction

Critical care therapy is dynamic and our knowledge about pathophysiology and treatment options is constantly expanding. However, on occasion, the emerging evidence also questions the effectiveness of long-used routines [1]. The pulmonary artery catheter (PAC), lung recruitment, daily chest X-rays, intensive renal replacement therapy, ‘supranormal’ haemodynamic targets, hypothermia, early total parental nutrition, and even protocols such as early goal-directed therapy have become outdated. Whereas, at least in the United States, overall mortality in intensive care units (ICUs) has decreased over time [2], the mortality of diseases commonly seen in ICU, such as sepsis—the leading cause of death in hospitalised patients—has shown no significant decrease in mortality over time [3].

Through critical care research, there are two important lessons that have been learned: first, there are very few protocols that fit the majority of our patients, so care has to be individualised; and second, once a disease has taken hold, treatment is more difficult and often lacks the desired effectiveness, so greater emphasis has to be laid on prophylaxis or treatment in the very early stages of a condition.

For example, considering the administration of fluids, there is specific advice for particular situations—such as an initial bolus of up to 30 mL/kg in septic shock—that is backed up by current recommendations [4], but there is still no general protocol for fluid therapy applicable in many other common ICU conditions [5,6]. The reason for this lies in the heterogeneity of the patients, even if they display the same symptoms and suffer from identical disease states. A patient with diastolic dysfunction and aortic stenosis reacts differently to a fluid bolus than a patient with pulmonary hypertension (PH) and severe mitral regurgitation. This reveals a significant problem in our daily work; that is, patients present with multiple underlying conditions that are increasingly complex and interact with each other. As we often have only low-grade evidence for the treatment of even common diseases, there is limited to no evidence when it comes to very complex scenarios.

In addition to using all available evidence, our department also extensively uses ultrasonography of the respiratory system, general critical care ultrasound (GCCUS), and critical care echocardiography (CCE) to evaluate our patients and aid daily decision-making [7,8,9,10]. Our experience with these methods helps us to guide treatment in a variety of scenarios and to treat our patients in an individualised manner. Most importantly, ultrasonography and echocardiography not only help us to evaluate our patients once only, they allow us to re-evaluate our patients repeatedly, to refine therapy [7]. These methods thus not only support us in what and how to treat, but also when to step back or stop active treatment.

The presented case vignettes aim to demonstrate the role of critical care ultrasonography for assessing respiratory function and haemodynamics in a variety of clinical settings in critically ill patients. Taken from a pool of prospectively enrolled and assessed study patients (participants of three different studies as described in detail in the next section), the five clinical scenarios highlight how ultrasonography altered clinical therapy. The clinical decision-making process for altering individual clinical management with ultrasound and echocardiographic data is outlined, which will assist other practitioners to understand how ultrasound and echocardiographic data can be used clinically to guide practical bedside medical management.

## 2. Materials and Methods

### 2.1. Patients, Study Setting, and Institutional Approval

In this single-arm retrospective case-based report, representative complex clinical ICU scenarios in which ultrasonography considerably altered or guided management were randomly identified from recently published studies on focused assessment of sonographic pathologies in the intensive care unit (FASP-ICU study) [7] and critical care echocardiography [8,9], as well as a further so far unpublished study. The clinical decision-making process for therapy changes due to ultrasound and echocardiographic evaluations and re-evaluations is outlined.

The study was conducted in two surgical ICUs of a tertiary university hospital. A single expert operator trained in internal medicine and cardiology as well as anaesthesiology and intensive care undertook all ultrasound and echocardiographic examinations. A second reviewer was initially blinded and verified all diagnoses. Agreement was reached by handling disputes according to a pre-agreed protocol. Our local ethics committee waived the requirement for consent and approved retrospective anonymised data analysis (ethics proposal Universitaetsmedizin Goettingen [UMG] 14/9/15, 28 September 2015).

### 2.2. Echocardiographic Technique and Calculation of Haemodynamics

CCE was performed using standardised echocardiographic views according to guidelines of the American Society of Echocardiography [11,12,13]. Deviating from the guidelines [14], right ventricular (RV) dysfunction was evaluated according to tricuspid annular plane systolic excursion (TAPSE) measurements and defined as severe (<13 mm), moderate (14–16 mm), or mild (<17 mm). Non-invasive haemodynamic CCE and haemodynamic profiling was performed as previously described [9]. In brief, cardiac output (CO) and stroke volume (SV) were determined as follows: CO = (SV·HR)/1000; SV = π·(LVOT/2)^2^·VTI_LVOT_ [15] (HR: heart rate; VTI_LVOT_: left ventricular outflow tract velocity time integral). Systemic vascular resistance (SVR) was calculated according to the formula SVR = (80 [MAP − CVP])/CO (MAP: mean arterial pressure; CVP: central venous pressure). The Nagueh formula was used for the determination of mean pulmonary capillary wedge pressure (PCWP) (PCWP = 1.24·[E/average e’] + 1.9) [16]. For pulmonary vascular resistance (PVR), the Abbas equation was used: PVR = TRV/VTI_RVOT_·10 + 0.16 [17] (TRV: tricuspid regurgitation velocity; RVOT: right ventricular outflow tract). For calculation of the body surface area (BSA), the Dubois formula (BSA = 0.007184·W^0.425^·Ht^0.725^) was used.

### 2.3. Ultrasound Techniques

GCCUS was performed as previously described in detail [7].

Vascular Ultrasound: A linear probe was used in a compression sonography technique [18] at multiple sites of the vessel in two axes to diagnose thrombosis.

Pulmonary Ultrasound: A linear probe with most filters disabled was used for lung ultrasonography, which was carried out according to international recommendations [19] and practice recommendations [20] in the upper and lower anterior and lateral chest zones. Interstitial syndrome (presence of B-lines) and pulmonary oedema (bilateral B-pattern) were diagnosed in conjunction with CCE and GCCUS results and after alternative diagnoses were excluded [21].

### 2.4. Ultrasound Machine

A General Electric (GE) Healthcare Vivid S5 (GE Healthcare GmbH, Solingen, Germany) ultrasound machine, equipped with a phased array adult sector scanner (1.5–3.6 MHz), a linear array linear scanner (6.0–13.0 MHz), and a curved array convex scanner (1.8–6.0 MHz), was employed. All necessary Doppler capabilities (CD, CW, PW, and TD), imaging modalities (2D and motion-mode), and software features were available. Images were stored digitally and immediately analysed.

### 2.5. Data Recording and Handling

Clinical data were automatically recorded by IntelliSpace Critical Care and Anesthesia PDMS software (ICCA, Philips Healthcare, Amsterdam, The Netherlands). Ultrasound data were handled with Microsoft Excel (version 2013, Redmond, WA, USA). Haemodynamic calculations were computed as described above using Microsoft Excel. Statistics software (OriginPro, version 9.2, OriginLab Corporation, Northampton, MA, USA) was employed for the visualisation of haemodynamic profiles.

## 3. Results

**Figure 1:**
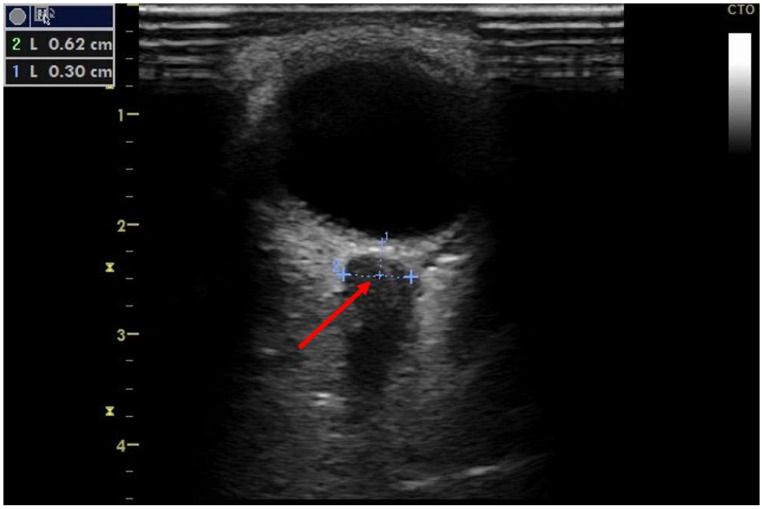
Ocular ultrasound in patient #1. Ocular ultrasound image. Optic nerve sheath diameter is measured 3 mm behind the globe at 6.2 mm (red arrow), indicating intracranial pressure above 20 mmHg.

Vignette #1: A 55-year-old gentleman was referred from a secondary hospital ICU to our unit for venovenous extracorporeal membrane oxygenation (VV-ECMO) for underlying acute respiratory distress syndrome (ARDS) of unknown origin. The patient had a history of rheumatoid arthritis with ongoing immunosuppressive therapy. Five weeks prior to referral, the patient underwent resection of a pulmonary squamous cell carcinoma. In the postoperative course, upper gastrointestinal bleeding from a duodenal ulcer occurred, causing haemorrhagic shock. He required percutaneous coronary intervention with the implantation of five cardiac stents into severely diseased coronary arteries, after an episode of ventricular fibrillation. Subsequent cardiogenic shock complicated and prolonged recovery. Continuing deterioration of pulmonary function and hypercapnia led to the referral to our institution. There were further complications, including development of abdominal compartment syndrome, which were treated accordingly. Every three to four days, the patient underwent whole-body GCCUS examination according to the FASP-ICU protocol [7] and daily non-invasive haemodynamic CCE [9]. Among other findings, ultrasound examination revealed an absence of lung sliding, consolidated lung, interstitial syndrome, pulmonary oedema, and a thrombus in the right internal jugular vein. Non-invasive haemodynamic assessment showed an SV of 74 mL (normal), a cardiac index (CI) of 2.54 L·min^−1^·m^−2^ (normal to mildly reduced), a systemic vascular resistance index (SVRI) of 2358 dyn·s·cm^−5^·m^−2^ (raised), an approximated PCWP of 13.8 mmHg (normal to mildly raised), and a systolic pulmonary artery pressure (sPAP) of 45 mmHg (significantly raised) with vasoactive support of 3 µg/min norepinephrine (NE). VV-ECMO flows were able to be continuously reduced, and pulmonary function improved significantly during the course of treatment. However, on day ten in our unit, an ultrasound examination of the diameter of the optic nerve sheath as part of our local protocol revealed intracranial pressure (ICP) above 20 mmHg (Figure 1). Despite the high risk of intrahospital transport for VV-ECMO patients, cranial computer tomography (CT) was conducted, which revealed an extensive left hemispheric intracranial bleed. A Codman microsensor ICP probe was inserted for monitoring and ICP readings exceeded 38 mmHg. Therapy was limited in accordance with the patient’s perceived wishes, and the patient died after withdrawal of active treatment.

**Figure 2:**
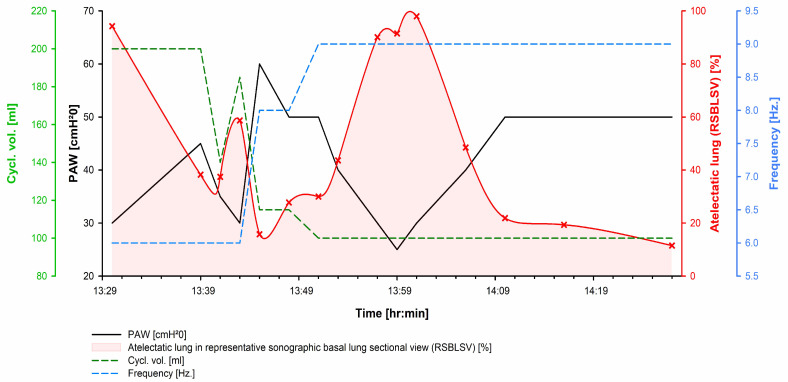
Atelectatic lung area during high-frequency oscillatory ventilation in patient #2. Cycle-volume, positive airway pressure, and the frequency of high-frequency oscillatory ventilation (HFOV) in relation to the atelectatic lung area (red, red shaded) estimated by lung sonography. An experimental HFOV pressure trial was performed over a duration of 30 min to reduce the atelectatic lung area and, consecutively, improve oxygenation. Time scale [hr:mm], Middle European Time (MET).

Vignette #2: A previously healthy 21-year-old gentleman was transferred from a secondary care hospital ICU to our unit for VV-ECMO treatment for underlying ARDS of unknown origin. The patient arrived ventilated via a pressure-controlled ventilation mode with a fraction of inspired oxygen (FiO_2_) of 0.7 and a positive end-expiratory airway pressure (PEEP) of 15 mmHg, resulting in a Horowitz index of 73. Despite lung-protective ventilation strategies, prone positioning, and an up-titration of the PEEP, respiratory function worsened and VV-ECMO treatment was initiated after CT imaging of the lung revealed pronounced bilateral consolidated lungs and fibrotic changes. Extubation was attempted, but failed because of hypoxia; partial pressures of oxygen in arterial blood (PaO_2_) were 50 mmHg after extubation. Pulmonary bleeding developed and could only be controlled after administration of blood coagulation factor XIII. PaO_2_ values subsequently dropped again to below 60 mmHg despite mechanical ventilation and ongoing VV-ECMO treatment; high-frequency oscillatory ventilation (HFOV) was thus initiated. Despite initial improvement in the hypoxia in response to HFOV, PaO_2_ values suddenly dropped again to 55 mmHg. Emergency ultrasonographic evaluation (extended GCCUS) revealed a malpositioned VV-ECMO inlet cannula in the right atrium, which was withdrawn immediately, and an almost completely consolidated lung (94%) with static air bronchograms. We performed an experimental HFOV recruitment manoeuvre by increasing the distension pressure from 25 mmHg to 60 mmHg. This resulted in a decrease in the atelectatic lung area from 94% to 16%. Lung recruitment had no lasting effect; the atelectatic lung area reached 91% within a few minutes. In response, we performed an experimental distension pressure trial under sonographic guidance (Figure 2). Based on this trial, we chose a distension pressure of 50 mmHg, a cycle volume of 100 mL, and an oscillator frequency of 9 Hz, settings we would not have chosen without direct sonographic visualisation. These settings resulted in a reduction in atelectatic lung area to about 11%; the PaO_2_ increased from 55 mmHg to 151 mmHg and reached 219 mmHg the next day, before the FiO_2_ and VV-ECMO flows were adjusted accordingly. Two additional mini-trials under sonographic guidance were performed, which showed that the effect of the prolonged recruitment manoeuvre persisted. A distension pressure of 35 mmHg at this stage resulted in an atelectatic lung area of about 13% (Figure S1). Haemodynamic changes were monitored by non-invasive echocardiography [9] and revealed that a 50 mmHg HFOV distension pressure resulted in diastolic dysfunction and an increase in PCWP and PVR (Table 1). However, sPAP fell and CI improved slightly, but the dose of the NE had to be more than doubled. On the third day after adjusting HFOV by sonographic evaluation, neurosonography revealed an increased ICP to more than 20 mmHg. An intracranial microtransducer probe was surgically inserted, with an initial ICP of 25 mmHg, and treatment was adjusted accordingly.

**Figure 3:**
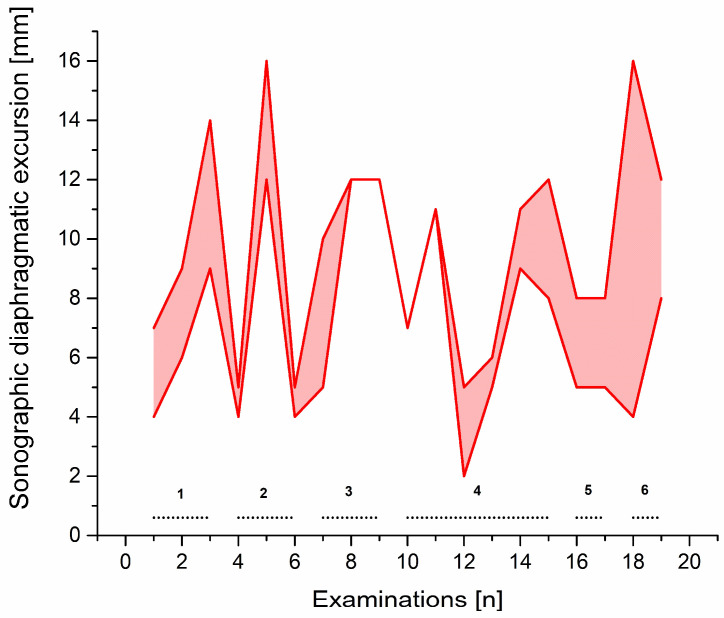
Diaphragmatic excursion during the six distinct weaning phases in patient #3. Diaphragmatic excursion measured by diaphragm sonography during a prolonged weaning period in a patient with Pompe disease. Upper red line depicts maximum diaphragmatic excursion and lower red line minimal diaphragmatic excursion during the examination period. The six distinct weaning phases and drawbacks are described in the main text.

Vignette #3: A 71-year-old gentleman with a lysosomal storage disorder (glycogen storage disease type II/Pompe disease) underwent coronary artery bypass grafting and—despite severe myopathy and impaired bilateral diaphragm function—initially only spent two days in our ICU before he could be discharged to a regular ward. Eighteen days later, the patient was urgently readmitted for uncontrolled atrial fibrillation, hypotension, and severe dyspnoea. Emergency intubation led to pulseless electrical activity and mechanical resuscitation resulted in return of spontaneous circulation (ROSC) after 20 min. Additional complications arose and were treated accordingly during the next days. However, liberation from mechanical ventilation failed despite the use of advanced weaning methods. The patient underwent insertion of a tracheostomy, but progress in weaning was still not achieved. We finally decided to try a neurally adjusted ventilatory assist (NAVA) ventilation weaning approach, in which the electrical diaphragm activity (Edi) is captured and synchronised with ventilation. Edi strength can be used as a parameter to assist and adjust liberation from mechanical ventilation. Although the weaning process made small progress and the patient showed improved tolerance towards this special mode of ventilation, Edi signals did not appear to be usable for weaning adjustments, owing to pre-existing nerve damage and myopathy. Sonographic diaphragm excursion measurements on multiple locations of the diaphragm were employed to guide weaning. Diaphragm excursion values measured by m-mode sonography proved to be the only parameter that correlated with the increasing respiratory fatigue reported by the patient. All conventional parameters stayed within normal ranges (Table S1). Weaning was further interrupted by complications, but the complications were able to be adequately assessed by sonographic diaphragmatic excursion and could thus be specifically addressed in the weaning process (Figure 3). The sonographically guided weaning process could be subdivided into six distinct phases: during phase 1, good weaning progress could be achieved with adjusting NAVA ventilation according to sonographic diaphragm excursion measurements, a negative fluid balance of 14 litres, and subsequently decreasing pleural effusions. However, at the end of phase 1, bleeding from the tracheostomy site developed and led to aspiration of blood. At the beginning of phase 2, the patient improved after the episode of aspiration and progress in weaning from ventilation could again be made. Time without ventilation could be increased until the middle of phase 2, where severe aspiration of food and the requirement for tube feeding once again set back the weaning process. The patient recovered from the aspiration in phase 3 and reached a stable plateau at the beginning of phase 4. Within phase 4, prolonged duration of spontaneous breathing resulted in a reduction in diaphragmatic excursion. Diaphragmatic excursion recovered during NAVA ventilation phases and, subsequently, the reduction in diaphragmatic excursion ceased to occur after periods of prolonged spontaneous breathing. The patient was now able to breath for prolonged periods (>6 h) without a tracheal cannula, but the weaning progress suffered a further setback by frequent panic attacks and depression (phase 5). After consulting psychotherapy and initiating treatment with opipramol, the tracheotomy was closed, and a mask continuous positive airway pressure home ventilation respirator was used during the night-time (phase 6). No further complications occurred and the patient was transferred to a regular ward after 67 days of ICU treatment and liberation from mechanical ventilation.

**Figure 4:**
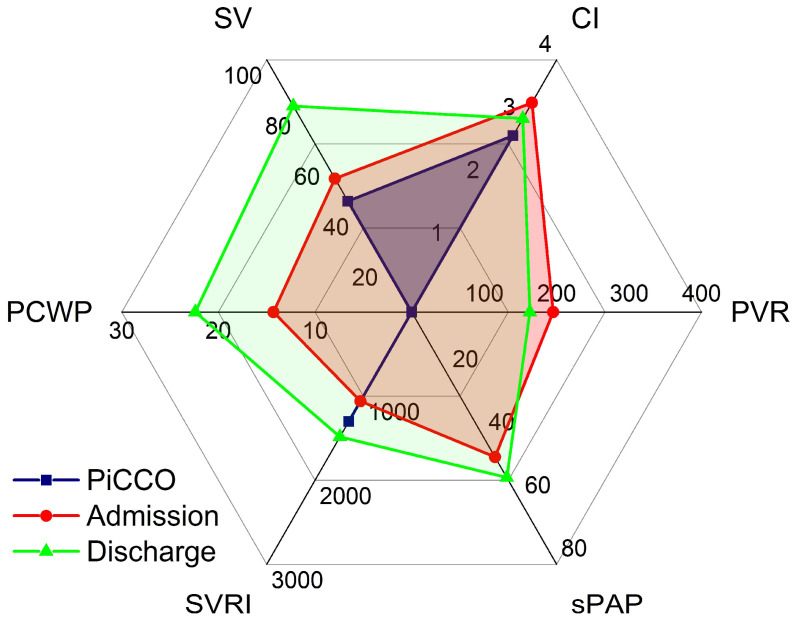
PiCCO misinterpreting significant tricuspid regurgitation as cardiogenic shock. Haemodynamic profiles on admission (red) and at discharge (green). Blue areas depict an inaccurate PiCCO measurement owing to significant tricuspid regurgitation. CI: cardiac index [L·min^−1^·m^−2^]; SV: stroke volume [mL]; PCWP: pulmonary capillary wedge pressure [mmHg]; SVRI: systemic vascular resistance index [dyn·s·cm^−5^·m^−2^]; sPAP: systolic pulmonary artery pressure [mmHg]; PVR: pulmonary vascular resistance [dyn·s·cm^−5^].

Vignette #4: A 74-year-old lady with a history of aortic stenosis, pulmonary hypertension (PH), and LV dysfunction (left ventricular ejection fraction (LVEF) of 30%) received a biological aortic replacement and was moved from our ICU to a regular ward 22 h postoperatively. Two days later, the serum potassium level was 7.8 mmol/L in a routine blood test and an ECG showed broad QRS complexes. The patient was re-admitted to our ICU; 2 g of calcium was administered intravenously, and a dual-lumen acute dialysis catheter was inserted into the left femoral vein for haemodialysis. During catheter insertion, the patient suddenly lost consciousness with ECG monitoring displaying asystole. ROSC was achieved after three minutes of cardiopulmonary resuscitation (CPR). Transoesophageal echocardiography (operator not certified) showed severely reduced LV and RV function. A PiCCO catheter was inserted, which measured a CI of 2.2 L·min^−1^·m^−2^. The patient was treated for cardiogenic shock including administration of dobutamine (DOB). One day later, expert CCE revealed severely reduced RV and moderately to severely reduced LV function in the presence of moderate to severe tricuspid regurgitation (TR). In the presence of TR, SV was 53 mL, CI was 3.32 L·min^−1^·m^−2^ (simultaneous PICCO CI 2.8 L·min^−1^·m^−2^, a difference of 16% at a heart rate of 116·min^−1^), SVRI was 1061 dyn·s·cm^−5^·m^−2^, PCWP was 14.3 mmHg, PVR was 195 dyn·s·cm^−5^, and sPAP was 46 mmHg (Figure 4). A thrombus in the right internal jugular vein and the left femoral vein (site of dialysis catheter insertion) was detected by vascular ultrasound. The CCE showed the diagnosis of cardiogenic shock to be incorrect and instead suggested a pulmonary embolus (PE) owing to a dislodged femoral vein thrombus during the dialysis catheter insertion; appropriate treatment for the PE was thus commenced, in place of the treatment for the misdiagnosed cardiogenic shock. The PICCO had misinterpreted the recirculation of the dye owing to the significant TR as a low cardiac output state, which had resulted in the initial incorrect diagnosis of cardiogenic shock. Sedation was stopped and the patient was extubated. Supper was eaten without assistance on the same day.

**Figure 5:**
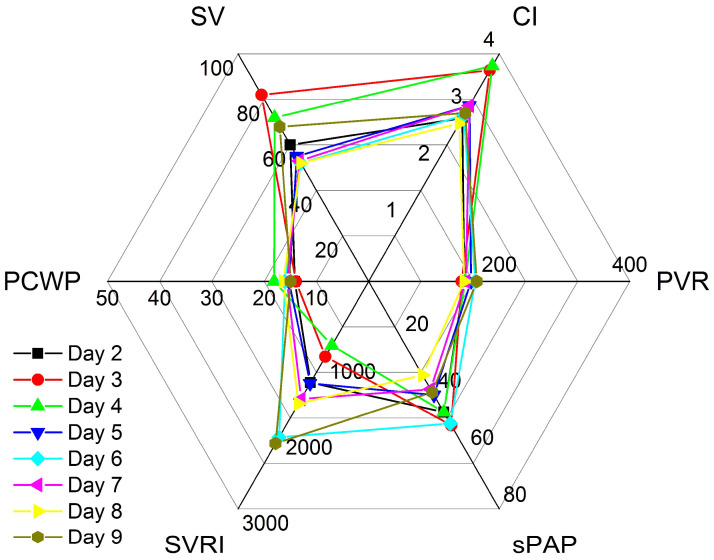
Haemodynamic profiling after heart transplantation. Haemodynamic profiles of patient #5 on different days of intensive care therapy. CI: cardiac index [L·min^−1^·m^−2^]; SV: stroke volume [mL]; PCWP: pulmonary capillary wedge pressure [mmHg]; SVRI: systemic vascular resistance index [dyn·s·cm^−5^·m^−2^]; sPAP: systolic pulmonary artery pressure [mmHg]; PVR: pulmonary vascular resistance [dyn·s·cm^−5^].

Vignette #5: A 59-year-old lady was admitted after heart transplantation (HTx) following a long history of dilated cardiomyopathy with an LVEF of 15%, PH, and recent malfunction of a CircuLite ventricular assist device. PAC readings on the first postoperative day compared excellently to echocardiographic non-invasive haemodynamic assessment and revealed good LV and RV function, second-degree diastolic dysfunction, a CI of 2.87 L·min^−1^·m^−2^, and an sPAP of 46 mmHg. The PAC was removed to avoid complications and further management was solely based on echocardiographic non-invasive haemodynamic measurements, which were combined to form profiles, as shown in Table 2 and Figure 5. On the third day, moderate RV dilatation developed and subsequently led to moderately reduced RV function in the presence of a hyperdynamic LVEF and a further sPAP increase to 51 mmHg. In response to haemodynamic values and pre-existing PH, the NE was reduced to lower the PVR and sPAP, but low-dose DOB was continued for RV support. On day four, moderate RV dilatation decreased to mild, but the RV function remained moderately reduced. Grade II diastolic dysfunction, an increased PCWP of 18.1 mmHg, and a low SVRI of 845 dyn·s·cm^−5^·m^−2^ necessitated further adjustment of the therapy (Table 2 and Figure 5). The SVRI decrease required higher doses of NE, while the high PCWP required higher doses of DOB. However, an increase in either NE or DOB led to reciprocal adverse effects. In our institution, we have good experience with discontinuing both agents whenever two competing factors arise. We thus reduced both the NE and DOB. Iloprost inhalational therapy was initiated to lower the PVR and improve RV function and low-dose isoprenaline initiated to improve the heart rate and prevent bradycardic episodes. In response, without DOB, the CI fell on day five but remained within a safe range, whereas diastolic function returned to normal, SVRI rose, PCWP fell, and sPAP and RV function slightly improved. Over the following days, the haemodynamics stabilised, RV function improved to mildly reduced, and sPAP values fell to as low as 33 mmHg. However, while the diastolic function worsened again to grade II dysfunction, the patient showed good physical performance during physiotherapy and was subsequently discharged from the ICU eight days following the HTx.

## 4. Discussion

Evidence to guide critical care therapy is continually expanding and, likewise, the number of recommendations and guidelines is increasing. Where available, scientific evidence should always be used first. However, therapy and disease states in intensive care are dynamic and constantly changing, which makes application of recommendations in ICU patients in some specific scenarios difficult or even impossible; this is especially the case when several disease states interact with each other or complications occur for which no primary evidence-based therapies are available.

The effectiveness of therapeutic measures can often only be broadly measured, such as the stabilisation of haemodynamics or improvement of laboratory values. When several aspects of therapy are adjusted simultaneously or during a short time, the effect of a particular action is often difficult to discern.

Ultrasonography and echocardiography offer a unique opportunity to visualise pathologies, abnormalities, and functional changes, for example in lung function or haemodynamics [7,8]. Therapeutic measures can thus be individualised, periodically re-evaluated, and adjusted based on effectiveness and adverse effects.

In addition to monitoring effectiveness, ultrasonography can also detect adverse effects or new clinical states that prompt alteration of therapy or identify disease states that may lead to the implementation of palliative treatment, such as the massive intracranial bleed in patient #1, despite improving lung function. In patient #2, a malpositioned VV-ECMO inlet cannula was detected by ultrasonography and subsequently corrected. Simultaneous with the identification of the mispositioned cannula, a nearly complete atelectatic lung was revealed. HFOV settings were adjusted accordingly, but to levels that would have been concerning without direct feedback from frequent ultrasonographic re-evaluations. While lung recruitment can be visualised by CT [22], several examinations during a short time period are better and more easily achieved by ultrasonography to avoid radiation exposure during CT and the need for time- and staff-consuming transport of ICU patients. Patient #2 also showed in a notable way that therapeutic changes must not only be monitored frequently in the same organ system, but can have additional adverse effects on other organ systems that make therapeutic re-adjustments necessary. In additional, repeated ultrasonography allows treatment to be adjusted regularly to optimise therapeutic effects while minimising adverse effects.

Whenever all conventional and established therapies fail, experimental approaches may be the last chance, such as in patient #3, when NAVA ventilation [23] was attempted after conventional weaning attempts had repeatedly failed. However, in the presence of underlying neurological disease, Edi values often cannot be interpreted according to standard protocols; in this patient, experimental sonographic monitoring of the diaphragm function [24] was used for the titration of the NAVA. In this unique setting, direct feedback of diaphragm activity assessed by ultrasonography was used to guide further therapy. This was the only modality that correlated with the respiratory fatigue mentioned by the patient. Neither guidelines nor recommendations were applicable for this situation, and only the combination of two experimental therapies led to successful liberation from mechanical ventilation after all conventional therapies had repeatedly failed.

Limitations of the classical haemodynamic monitoring tools (PAC and PiCCO) can dramatically incorrectly influence therapy decisions and diagnoses, as presented in patient #4, where significant TR led to inaccurate PiCCO measurements [25,26]. This repeatedly misguided the intensivist to initiate an incorrect therapy regime (treatment of cardiogenic shock instead of pulmonary embolism). At high heart rates, PAC and PiCCO measurements might only differ by 16% from actual haemodynamics, such as in patient #4, but may differ by around 30–50% at lower, more normal heart rates [9]. Initial focused GCCUS of patient #4 on admission would have detected the femoral vein thrombosis, thereby preventing detachment of the thrombus by the dialysis catheter, the pulmonary embolism, and the subsequent necessity for CPR.

The PAC has well-known limitations and, in addition, carries a mortality risk [27]. Despite this, the PAC is still in use today [28], partly because of the absence of suitable or easily available alternatives. Haemodynamic CCE for the first time offers a validated and non-invasive approach to obtain haemodynamic data [9]. The PAC is even now almost universally employed in patients after HTx owing to the high risk of RV failure. After ensuring that haemodynamic values (PVR, CI, and PCWP) derived by the PAC and CCE were comparable, patient #5 was managed solely by haemodynamic CCE. All haemodynamic values that could be obtained by the PAC were obtainable by CCE and these values were used to guide further therapy after RV compromise developed on day three. Use of vasopressor, catecholamine, and vasodilator therapy were solely based on the results from the haemodynamic CCE and haemodynamic profiling, as outlined in patient #5. When RV dysfunction develops, subsequent TR can lead to recirculation of the dye indicator and to false PAC/PiCCO measurements, as seen in patient #4 [25,26]. Haemodynamic CCE is not affected by TR and can measure additional variables such as parameters to evaluate diastolic function, and thus appears to be more useful and reliable than the PAC. Haemodynamic CCE values, such as a low CI, can now be attributed to clinical states, for example, ventricular failure or diastolic dysfunction, instead of solely registering deteriorating values. CCE offers the advantage of identifying the underlying pathology [8,9]. Specific treatment approaches can now be targeted to the underlying pathology.

Clinical states are often more complex than pathophysiological models and, when competing dysfunctions arise, such as in patient #5 with a low SVRI and a high PVRI, haemodynamic CCE is useful; in this case, CCE suggested discontinuing NE and DOB, a decision that we would not have made without the ultrasonography results.

Ultrasonographic techniques offer the advantage of diagnosing a vast spectrum of pathologies at the bedside and can be re-employed multiple times even on a daily basis to evaluate and especially to re-evaluate patients after therapeutic interventions [7,10].

## 5. Limitations of the Study

Haemodynamic CCE and GCCUS are not yet standards of care. Currently, in most countries, there are very few or no ultrasound operators even in tertiary and quaternary centres that perform sonographic and echocardiographic examinations with the full skill set and level demonstrated in this study. Even in our centre, a single expert operator, trained in cardiology as well as anaesthesiology and intensive care medicine, had to be employed for all ultrasonographic measurements and for adjusting therapies based on the results of sonographic evaluations. The single-operator approach can lead to obvious biases in data acquisition and interpretation. General limitations regarding the echocardiographic and ultrasound techniques employed in this study have been described in detail before [7,8,9]. Fortunately, as studies such as the current one demonstrate the usefulness of ultrasonography for clinical decision-making, ultrasound education is increasing around the world; operators skilled in critical care ultrasound are likely to become more widely available.

## 6. Conclusions

Compared with many bedside examination tools, ultrasonography of the respiratory system, GCCUS techniques, and haemodynamic CCE offers advantages in caring for critically ill patients in common ICU scenarios, but especially in the most complex scenarios, exceptional circumstances, and when experimental therapy approaches are used. The results from ultrasound examinations should not routinely be used to initiate treatment that deviates from established evidence and recommendations, but can be of immense help in scenarios for which no or only sparse primary evidence is available or in which this evidence cannot be applied owing to very complex disease interactions. Sonographic techniques can not only be used to reveal new abnormalities and guide further therapy, but can also contribute immensely to making the decision to transition from curative treatment to supportive care.

## Figures and Tables

**Table 1:** Assist device use, vasopressor therapy, and haemodynamic values in patient #2.

Day	BP	MAP	HR	CVP	NE	VV-ECMO	LV-EF	RV-fct.	Diastol.-fct.	SV	CI	SVRI	PCWP	PVR	sPAP
	mmHg	mmHg	per min	mmHg	µg/min	flow L				ml	L·min^−1^·m^−2^	dyn·s·cm^−5^·m^−2^	mmHg	dyn·s·cm^−5^	mmHg
7	91/44	58	93	20	8	5.5	Normal	Normal	Normal	60	2.92	1040	11.8	178	54
8	85/51	62	123	10	20	5	Normal	Normal	II°	49	3.21	1297	20.1	233	44
9	95/56	67	112	15	20	5	Normal	↓	II°	55	3.22	1292	13.5	156	46

ICU parameters, vasopressor therapy, venovenous ECMO flows, and non-invasive critical care echocardiographic (CCE) and vital parameters of patient #2. BP: blood pressure; HR: heart rate; CVP: central venous pressure; NE: norepinephrine; VV-ECMO: venovenous ECMO; LVEF: left ventricular ejection fraction; RV-fct.: right ventricular function; Diastol. fct.: diastolic function; SV: stroke volume; CI: cardiac index; SVRI: systemic vascular resistance index; PCWP: pulmonary capillary wedge pressure; PVR: pulmonary vascular resistance; sPAP: systolic pulmonary artery pressure. ↓: mildly reduced.

**Table 2:** Haemodynamics and vasoactive and inotropic drug therapy after heart transplantation in patient #5.

Day	BP	MAP	HR	CVP	PAC	PAC CI	PAC PCWP	NE	DOB	INN	Iloprost	Sildenafil	LV-EF	RV-fct.	Diast.-fct.	SV	CI	SVRI	PCWP	PVR	sPAP
	mmHg	mmHg	per min.	mmHg	mmHg	L·min^−1^·m^−2^	mmHg	µg/min	µg/kg/min	µg/min	µg	mg				ml	L·min^−1^·m^−2^	dyn·s·cm^−5^·m^−2^	mmHg	dyn·s·cm^−5^	mmHg
2	100/50	65	80	17	43/20 (30)	2,8	15	7	4	0			Normal	Normal	II°	60	2.87	1336	14.2	146	46
3	109/50	68	76	22	removed	removed	removed	7	4	0			↑	↓↓	Normal	82	3.71	993	13.9	142	51
4	95/40	56	88	16	-	-	-	3	4	0.33	3 × 5 µg		Normal	↓↓	II°	72	3.79	845	18.1	148	46
5	113/45	64	94	12	-	-	-	0	1	0.33	6 × 5 µg		Normal	↓↓	Normal	55	3.09	1345	15.3	157	40
6	153/73	95	94	29	-	-	-	0	0	0.33	6 × 5 µg		Normal	↓↓	I°	52	2.92	2056	16	163	50
7	116/45	66	98	6	-	-	-	0	0	0.33	6 × 5 µg		Normal	↓↓	I°	53	3.09	1552	15.6	149	38
8	92/54	64	90	8	-	-	-	0	0	0	3 × 5 µg	2 × 10 mg	Normal	↓↓	II°	52	2.78	1612	16.3	146	33
9	106/60	87	73	8	-	-	-	0	0	0	-	-	Normal	↓	II°	68	2.96	2138	14.9	166	39

ICU parameters, pharmacological therapy, PAC values, and non-invasive echocardiographic haemodynamic parameters (CCE) of patient #5. BP: blood pressure; MAP: mean arterial pressure; HR: heart rate; CVP: central venous pressure; PAC: pulmonary artery catheter (systolic/diastolic (mean) pressures); NE: norepinephrine; DOB: dobutamine; INN: isoprenaline; LV-EF: left ventricular ejection fraction; RV fct.: right ventricular function; Diastol. fct.: diastolic function; SV: stroke volume; CI: cardiac index; SVRI: systemic vascular resistance index; PCWP: pulmonary capillary wedge pressure; PVR: pulmonary vascular resistance; sPAP: systolic pulmonary artery pressure; ↑ hyperdynamic; ↓: mildly reduced; ↓↓: moderately reduced.

## Data Availability

The data presented in this study are available on request from the corresponding author. The data are not publicly available due to privacy and ethical restrictions.

## Supplementary Materials

The following supporting information can be downloaded at https://www.mdpi.com/article/10.3390/clinpract12060102/s1. Figure S1: Atelectatic lung area during high frequency oscillatory ventilation follow-up. Table S1. Selected weaning overview of patient #3.
