# Supplementary material for: Clinical Decision-Making in Practice with New Critical Care Ultrasound Methods for Assessing Respiratory Function and Haemodynamics in Critically Ill Patients
Source: Clin Pract. 2022 Nov 25;12(6):986–1000. doi: 10.3390/clinpract12060102 (PMC9776659; doi:10.3390/clinpract12060102)
Supplement: Supplementary file 1 [file clinpract-12-00102-s001.zip › clinpract-1924898 Table S1.pdf]

Table S1 **Selected weaning overview of patient #3**

Day Exam.	Time hr:mm	Vent.-mode	Diaph.-excur. mm	Change to	PSV FiO_2	NAVA FiO_2	PSV PEEP	NAVA PEEP	PSV ΔASB	NAVA ΔASB	PSV P_{max}	NAVA P_{max}	PSV P_{mean}	NAVA P_{mean}	PSV Fn./min.	NAVA Fn./min.	PSV $Edi_{min.}$	NAVA $Edi_{min.}$	PSV $Edi_{max.}$	NAVA $Edi_{max.}$	PSV MV	NAVA MV	PSV Vt	NAVA Vt
15	12:21	NAVA	4 - 7	PSV → NAVA	0.35	0.35	8	10	10	3	20.5	17	11.4	11.7	28	18	0.48	0.12	1.9	3.9	8.1	4.9	307	595
16 (1)	14:07	PSV	6 - 9	PSV → NAVA	0.5	0.5	10	10	6	7	17.4	18	12.6	12.3	27	22	23.8	0.16	37.5	4.8	7.8	7.7	344	369
16 (2)	17:36	NAVA	9 - 14	NAVA (2)	-	0.5	-	10	-	7	-	15.9	-	12.9	-	24	-	1.34	-	2.6	-	9.7	-	-
17 (1)	11:03	PSV	4 - 5	PSV → NAVA	0.3	0.3	10	10	7	7	17.6	17.4	11.9	11.7	20	23	2.54	0.32	5.9	2.9	8.5	6.7	410	209
17 (2)	14:05	NAVA	12 - 16	NAVA (2)	-	0.3	-	10	-	7	-	17.9	-	12.8	-	21	-	0.44	-	4.8	-	7.5	-	418
17 (3)	17:26	Spont. trach.	4 - 5	-	-	-	-	-	-	-	-	-	-	-	-	-	-	-	-	-	-	-	-	-
21	13:57	NAVA	5 - 10	-	-	0.3	-	6	-	7	-	15.4	-	9.1	-	24	-	0.2	-	4.9	-	7.6	-	303
29	16:05	PSV	12	-	0.45	-	6	-	12	-	19	-	10	-	22	-	-	-	-	-	8.1	-	308	-
30 (1)	12:07	PSV	12	PSV → NAVA	0.45	0.45	8	8	15	7	23.8	16	10.6	9.1	17	17	-	0.4	-	1.7	5.5	8.5	298	546
30 (2)	12:27	Spont.	7	-	-	-	-	-	-	-	-	-	-	-	-	-	-	-	-	-	-	-	-	-
32 (1)	08:08	NAVA	11	-	-	0.45	-	8	-	7	-	18.3	-	10.1	-	17	-	0.16	-	5	-	6	-	411
32 (2)	08:58	Spont. trach.	2 - 5	-	-	-	-	-	-	-	-	-	-	-	-	-	-	-	-	-	-	-	-	-
32 (3)	14:03	NAVA	5 - 6	-	-	0.3	-	8	-	7	-	13	-	9.5	-	22	-	0.16	-	4.9	-	7.4	-	241
32 (4)	14:30	Spont. trach.	9 - 11	-	-	-	-	-	-	-	-	-	-	-	-	-	-	-	-	-	-	-	-	-
40	14:02	BIPAP	8 - 12	BIPAP → NAVA	0.4	0.4	8	8	8	8	17.2	13.5	10.5	9.3	26	18	0.42	0.12	1.1	3.6	7.2	5.5	232	276
43	14:41	Spont. trach.	5 - 8	-	-	0.4	-	8	-	20	-	13.8	-	9.7	-	12	-	0.22	-	4.8	-	4.1	-	292
47	15:36	Spont. trach.	5 - 8	-	-	0.6	-	7	-	20	-	16.5	-	9.8	-	23	-	0.16	-	4.7	-	8.1	-	308
63	12:07	Spont.	4 -16	-	-	-	-	-	-	-	-	-	-	-	-	-	-	-	-	-	-	-	-	-
64	15:31	Spont.	8 - 12	-	-	-	-	-	-	-	-	-	-	-	-	-	-	-	-	-	-	-	-	-

Selected ventilatory modes, neurally adjusted ventilatory assist ventilation and phases of spontaneous or supported breathing over time during a prolonged weaning period from a mechanical ventilator in a patient with Pompe Disease.
