# Supplementary figures and images for: Clinical Decision-Making in Practice with New Critical Care Ultrasound Methods for Assessing Respiratory Function and Haemodynamics in Critically Ill Patients
Source: Clin Pract. 2022 Nov 25;12(6):986–1000. doi: 10.3390/clinpract12060102 (PMC9776659; doi:10.3390/clinpract12060102)

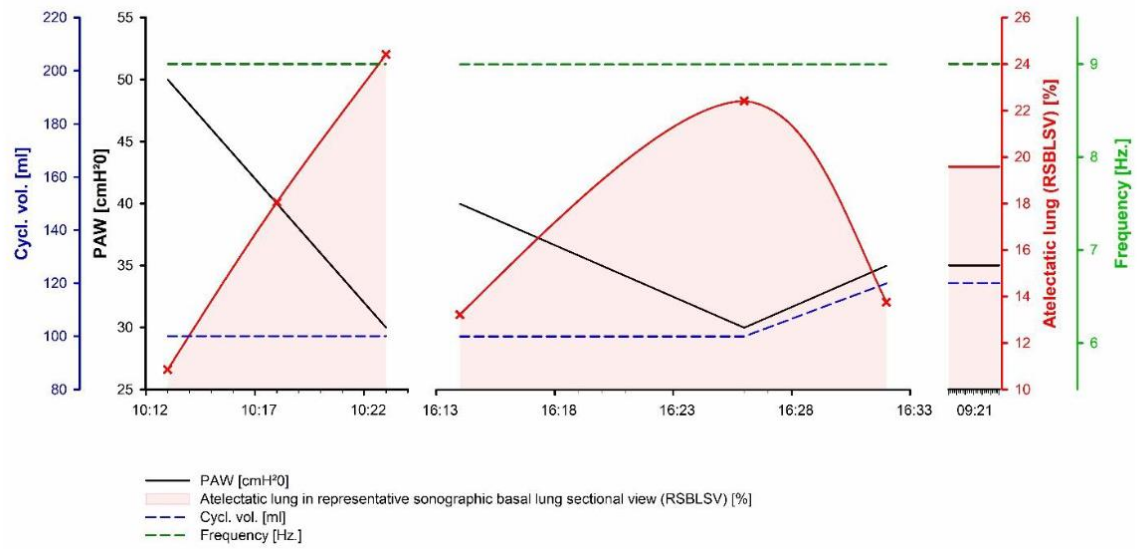

**Figure S1.** Atelectatic lung area during high frequency oscillatory ventilation follow-up.

Supplement: Supplementary file 1 [file clinpract-12-00102-s001.zip › clinpract-1924898 Figure S1.pdf]
